# Characterization of basal ganglia volume changes in the context of HIV and polysubstance use

**DOI:** 10.1038/s41598-022-08364-0

**Published:** 2022-03-14

**Authors:** Andrew J. Monick, Michelle R. Joyce, Natasha Chugh, Jason A. Creighton, Owen P. Morgan, Eric C. Strain, Cherie L. Marvel

**Affiliations:** 1 Sidney Kimmel Medical College, Thomas Jefferson University, Philadelphia, PA 19107 USA; 2 Department of Neurology, Johns Hopkins University School of Medicine, 1620 McElderry St., Reed Hall W102A, Baltimore, MD 21205 USA; 3 Department of Psychology, Cornell University, Ithaca, NY 14853 USA; 4 Department of Psychiatry and Behavioral Sciences, Johns Hopkins University School of Medicine, Baltimore, MD 21205 USA

**Keywords:** Magnetic resonance imaging, Addiction, HIV infections, Neurological manifestations

## Abstract

HIV and psychoactive substances can impact the integrity of the basal ganglia (BG), a neural substrate of cognition, motor control, and reward-seeking behaviors. This study assessed BG gray matter (GM) volume as a function of polysubstance (stimulant and opioid) use and HIV status. We hypothesized that comorbid polysubstance use and HIV seropositivity would alter BG GM volume differently than would polysubstance use or HIV status alone. We collected structural MRI scans, substance use history, and HIV diagnoses. Participants who had HIV (HIV +), a history of polysubstance dependence (POLY +), both, or neither completed assessments for cognition, motor function, and risk-taking behaviors (*N* = 93). All three clinical groups showed a left-lateralized pattern of GM reduction in the BG relative to controls. However, in the HIV + /POLY + group, stimulant use was associated with increased GM volume within the globus pallidus and putamen. This surpassed the effects from opioid use, as indicated by decreased GM volume throughout the BG in the HIV-/POLY + group. Motor learning was impaired in all three clinical groups, and in the HIV + /POLY + group, motor learning was associated with increased caudate and putamen GM volume. We also observed associations between BG GM volume and risk-taking behaviors in the HIV + /POLY- and HIV-/POLY + groups. The effects of substance use on the BG differed as a function of substance type used, HIV seropositivity, and BG subregion. Although BG volume decreased in association with HIV and opioid use, stimulants can, inversely, lead to BG volume increases within the context of HIV.

## Introduction

Substance use presents a significant health concern among populations affected by human immunodeficiency virus (HIV). A recent review estimated that among an estimated 15.6 million injection drug users globally, 17.8% live with HIV infection^1^. Among those who injected drugs, 82.9% specified opioids as a drug of choice and 33.0% reported stimulant use (with or without opioids). Drug use impacts disease progression among people living with HIV (PLWH)^2,3^ and elevates the risk of viral transmission through reduced adherence to antiretroviral therapy (ART) regimen^4^, more frequent unprotected penetrative sex^5,6^, and needle-sharing^7^. Despite the high proportion of PLWH who use substances and those substances’ effect on viral pathogenesis, the effects of substance use on the brain of HIV + individuals are ill-understood.

HIV degrades the permeability of the blood–brain barrier (BBB)^8^. Once inside the brain, the virus preferentially targets the basal ganglia (BG)^9,10^, leading to volumetric reduction among the caudate, putamen, and globus pallidus^9,11–14^. HIV has also been associated with other structural and functional changes to the BG, such as inflammation-related increases in white matter (WM)^15^ and disruption of frontostriatal circuits^16^. Underlying these changes to the BG in the early stages of HIV is the destruction of dopaminergic neurons^10^.

BG damage carries consequences for the motor, cognitive, and reward systems^9,17–22^. At least some of these effects are due to dopaminergic deficits^23–25^*.* Stimulants, including cocaine and methamphetamine, alter dopaminergic systems and appear to have mixed effects on the BG. Some studies have reported BG volume increases associated with stimulant use (e.g., due to neuroinflammation)^17–22^. Jernigan et al.^22^, for instance, found that methamphetamine dependence was associated with volumetric gain in the BG and parietal cortex. The authors postulated that this change may be attributed to physiological processes spurred by stimulant use that secondarily increase BG volume, such as glial activation and neuritic growth. Still other studies have reported stimulant-associated volumetric decreases in the BG. Among participants who reported long-term cocaine use, Barros-Loscertales et al.^23^ noted lower gray matter (GM) volume in the striatum, while Gardini & Venneri^24^ found lower GM volume in the right putamen and insula relative to controls. Opioid use, by contrast, seems to consistently lead to BG GM reductions in the caudate, putamen, and GP^25–27^. Given the strength of these independent effects, it is likely that the concomitant use of stimulants and opioids may exert complex effects over the dopaminergic system and the BG in PLWH.

The combined pathogenic effects on the BG from both HIV and psychoactive substances remain largely unknown. To our knowledge, only one study has examined BG volume in substance-dependent populations as a function of HIV^22^. Independent effects of methamphetamine were associated with increased GM; independent effects of HIV were associated with decreased GM. Effects from combined methamphetamine use and seropositivity were not observed, suggesting that opposing effects masked GM changes in the BG. Alterations to the BG among PLWH who use opioids, in contrast, have not been examined^28^. It thus remains unclear whether the BG is differentially affected by stimulants and/or opioids within the context of HIV infection.

In this study, we examined the effects of HIV infection on BG GM with respect to real-world polysubstance use (defined as stimulant and opioid use). We hypothesized that protracted polysubstance use, comprising primarily opioids and stimulants, would be associated with BG changes in GM in PLWH. Such changes could manifest as volume decrease (e.g., neuronal atrophy), volume increase (e.g., neuroinflammation), or no net change (both factors interacting to mask effects). We also explored the relation between BG structural changes as a function of HIV and polysubstance use and cognition, motor function, and risk-associated behavior.

## Methods and materials

### Participants

#### Recruitment and demographics

Participants were seropositive and/or endorsed a history of polysubstance dependence. Recruitment included referrals from an HIV Neurology Service and HIV Dementia studies, flyer postings, and local methadone clinics. This study included four groups: (1) HIV-negative persons with no history of polysubstance dependence (HIV-/POLY-); (2) HIV-negative persons with a history of polysubstance dependence (HIV-/POLY +), (3) HIV-positive persons with no history of polysubstance dependence (HIV + /POLY-), and (4) HIV-positive persons with a history of polysubstance dependence (HIV + /POLY +) (See Table 1 for demographics).

**Table 1:** Demographics of the four study groups (*N* = 93).

	HIV-/POLY− (*n* = 34)	HIV− /POLY + (*n* = 27)	HIV + /POLY− (*n* = 17)	HIV + /POLY + (*n* = 15)	*P*-value
Age years (SD)	50.92 (12.6)	46.09(11.8)	57.28 (9.0)	56.53 (4.3)	0.129^A-B^, **0.045**^A-C^, 0.100^A-D^, **0.001**^B-C^, **0.002**^B-D^, 0.765^C-D^
Sex M:F	19:15	15:12	16:1	9:6	0.980^A-B^, **0.006**^A-C^, 0.788^A-D^, **0.006**^B-C^**,** 0.780^B-D^, **0.020**^C-D^
Education years (SD)	14.37 (2.1)	12.14 (1.5)	15.06 (2.0)	13.07 (2.2)	** < 0.001**^A-B^, 0.258^A-C^, 0.062^A-D^, < **0.001**^B-C^, 0.159^B-D^, **0.012**^C-D^
Handedness R:L:A	30:1:3	24:1:2	14:3:0	11:2:2	N.S
RAB (SD)	8.00 (4.7)	26.84 (13.7)	9.14 (3.1)	23.14 (11.7)	**0.001**^A-B^, 0.951^A-C^, < **0.001**^A-D^, < **0.001**^B-C^, 0.842^B-D^, < **0.001**^C-D^
BIS-11 (SD)	61.00 (16.5)	89.64 (11.3)	66.47 (13.3)	80.60 (19.6)	**0.002**^A-B^, 0.573^A-C^, **0.002**^A-D^, **0.002**^B-C^, 0.837^B-D^, **0.011**^C-D^

When a variable contained data from all 4 groups, an ANOVA (or Pearson Chi-Square for categorical variables) was performed first in order to determine significant group differences. Statistics from post-hoc comparison are reported in the table as follows: A = HIV-/POLY-, B = HIV-/POLY + , C = HIV + /POLY-, D = HIV + /POLY + . Group comparisons are indicated by the two letters as marked (e.g., A-B indicates Group A vs. Group B). N.S. = not significant, per original omnibus test and no post-hoc comparisons were conducted; SD = standard deviation, M:F = male: female, R:L:A = right: left: ambidextrous. Boldface indicates significance, *p* < .05.

Participants were screened for Axis I psychiatric disorders using the abbreviated Structured Clinical interview for DSM-IV AXIS I (SCID)^29^ and excluded for a history of depression that was unrelated to substance use, psychotic disorders, post-traumatic stress disorder, or anxiety disorders. The SCID was also used to determine substance dependence. Participants who endorsed dependence upon cocaine and/or opioids were enrolled. Although some participants had used stimulants recreationally, they did not meet criteria for dependence and were placed in a POLY- group. Opioid-dependent participants who were receiving opioid maintenance therapy at the time of testing were treated with either methadone or buprenorphine for at least 2 months prior to the MRI. A modified Lifetime Drug Usage Questionnaire^30–33^ was used to quantify lifetime substance exposure. All participants were abstinent from illicit substances for at least 60 days prior to the MRI (per verbal report). Those who were undergoing opioid therapy provided urine drug screen results from their clinic for additional confirmation of drug-negative testing for the prior 60 days.

Urine drug testing occurred during pre-screening visits about one week prior to the MRI and again on the day of the MRI scan for the following substances: cocaine, amphetamine, methamphetamine, marijuana, methadone, opiates, phencyclidine, barbiturates, and benzodiazepines (Germaine Laboratories- 9 version Aimscreen™ Multi-Drug Urine Test DipDevice). Blood alcohol level was tested via breathalyzer. HIV serostatus was confirmed through blood testing or verified by medical record review.

Participants were excluded if they had a history of neurological conditions (other than HIV), head injury resulting in prolonged loss of consciousness (> 5 min) and/or neurological sequelae, presence of untreated or uncontrolled chronic psychiatric illness, severe or unstable medical disorder, uncontrolled high blood pressure, hepatitis C status requiring immediate medication, pregnancy, or metal fragments or implants within the body.

Initially, 100 participants were enrolled in the study. Seven participants were excluded for a history of alcoholism (HIV-/POLY + , *n* = 5), positive urine drug test on the day of the scan (HIV-/POLY + , *n* = 1), and incidental finding on the MRI (HIV-/POLY + , *n* = 1). Thus, the final number of participants was *N* = 93: HIV-/POLY- (*n* = 34), HIV-/POLY + (*n* = 27), HIV + /POLY- (*n* = 17), and HIV + /POLY + (*n* = 15).

This study was approved by the Johns Hopkins Medicine Institutional Review Board. All methods were performed in accordance with the Declaration of Helsinki. All patients provided written informed consent.

### HIV clinical data

HIV-related clinical information was abstracted from participants’ medical records: duration of HIV infection, prior AIDS diagnosis (CD4^+^ T-cell count ≤ 200 cells/mm^3^)^34^, current (within 6 months) CD4^+^ T-cell count and HIV viral load CD4 nadir, and exposure to efavirenz (EFV) antiretroviral medication (see Table 2). We expanded search parameters for three participants who did not have CD4 values available within 6 months of the visit to a measurement within 5 years (HIV + /POLY-, *n* = 2; HIV + /POLY + , *n* = 1). Undetectable viral loads were defined as < 20 copies/mL^35^. Three participants surpassed this threshold (HIV + /POLY- (36 copies/mL); HIV + /POLY + (23 and 97 copies/mL), but met criteria for viral suppression (defined as less than 200 copies/mL)^35–37^.

**Table 2:** Clinical variables across groups.

	HIV-/POLY− (*n* = 34)	HIV−/POLY + (*n* = 27)	HIV + /POLY− (*n* = 17)	HIV + /POLY + (*n* = 15)	*P*-value
Estimated duration of HIV infection years (SD)	–-	–-	21.53 (8.7)	21.27 (8.1)	0.930
AIDS (CD4 < 200 cells/mm^3^)	–-	–-	41.2	73.3	0.067
Current CD4 count (SD)	–-	–-	762.18 (304.2)	642.60 (314.0)	0.284
CD4 nadir (SD)	–-	–-	322.65 (282.8)	133.07 (204.6)	**0.037**
HIV viral load (n < 20 copies/mL)	–-	–-	15	13	0.471
Current or past exposure to EFV (n)	–-	–-	11	11	0.779
ART adherence lifetime (SD)	–	–	92.82 (9.8)	84.40 (13.3)	0.054

Statistics are reported as in Table 1. SD = standard deviation. Significant values are in [bold], *p* < 0.05.

### Neuroimaging

#### Image acquisition

MRI scans were performed using a 3.0 Tesla Philips scanner (Intera, CX, Achevia, and Elition models) and 32-channel head coil. Structural images were collected using a sagittal magnetization prepared gradient-echo (MPRAGE) sequence: repetition time /echo time = 6.9/3.3 ms; field of view = 240 × 240; 170 slices; slice thickness 1.0 mm; 0 mm gap; flip angle = 8 degrees; voxel size = 0.75 × 0.75 × 1.0 mm. Total scan duration was 6 min.

#### Image preprocessing

T1-weighted images were preprocessed following the voxel-based morphometry pipeline^38^ within Statistical Parametric Mapping version 12 (SPM12)^39^ using MATLAB R2020b^40^. Images were reoriented to the anterior commissure and segmented into tissue classes. Individual templates were created from the GM segmentation images with diffeomorphic anatomical registration through exponentiated lie algebra (DARTEL) toolbox, warped into common Montreal Neurological Institute (MNI) space, Jacobian-scaled to estimate GM intensity, and smoothed with an 8 mm full-width at half-maximum (FWHM) kernel. Regions of interest (ROI) masks were coregistered and resliced to GM segmentation images (voxel size 1.5mm^3^).

#### Neuroimaging analyses and statistics

Total GM, WM, and cerebrospinal fluid (CSF) volumes were calculated using MATLAB get_totals script (http://www0.cs.ucl.ac.uk/staff/g.ridgway/vbm/get_totals.m) in SPM12. We summed these tissue volumes (GM, WM, and CSF) for total intracranial volume (TCV) values. GM volumes were similarly computed for the following ROIs: total BG, caudate, globus pallidus external (GPe), globus pallidus internal (GPi), putamen, subthalamic nucleus, substantia nigra pars compacta (SNc), substantia nigra pars reticulata (SNr), ventral tegmental area (VTA), and nucleus accumbens (NAc) for left and right hemispheres^41,42^. Two-sampled t-tests were performed to analyze voxelwise group GM differences in total BG and ROIs between the HIV-/POLY- (controls) versus each of the three clinical groups. Multiple regression analyses were used to assess the relationship between GM volume in total BG and ROIs for the duration of opioid/stimulant use and opioid/stimulant frequency of use. Separate regression analyses were performed to examine motor learning and risk-associated behaviors. For t-tests and regressions, age, sex, education, TCV, and handedness were included as covariates. Whole-brain GM was used as an additional covariate in the between-group comparisons. For these GM measures, a threshold of *p* < 0.05 family-wise error (FWE) was used, and significant data from cluster sizes of ≥ 5 voxels were reported.

### Task procedures

#### Behavioral assessments

##### Risk assessment battery (RAB)

The Risk Assessment Battery (RAB)^43^ is a 45-item, self-administered questionnaire comprised of two sub-scales of activity (drug and sex risk items) that have occurred within the past 30 days. Scores are combined to calculate a total for risky behaviors, with a maximum high-risk score of 40. [HIV-/POLY- (*n* = 17), HIV-/POLY + (*n* = 14), HIV + /POLY- (*n* = 15), and HIV + /POLY + (*n* = 14)].

##### Barratt impulsivity test (BIS-11)

The Barratt Impulsivity Test (BIS-11) is a 30-item, self-administered questionnaire that assesses impulsive behavior^44^. A total score was computed with a range of 30–120, where higher scores indicate greater impulsivity [HIV-/POLY- (*n* = 33), HIV-/POLY + (*n* = 24), HIV + /POLY- (*n* = 16), and HIV + /POLY + (*n* = 15)].

##### Motor and cognitive tasks

A subset of participants received motor, cognitive, and dual task paradigms following Kronemer and colleagues^45,46^. (See also Supplementary Information for method details and group sample sizes per task). Participants performed a motor task that involved continuous drawing of “figure-8 s”, a cognitive task that involved working memory for letter sequences, and a combined motor-cognitive task that involved performance of both tasks simultaneously. Our primary outcomes of interest involved group effects and/or interactions.

### Statistical analyses of behavioral, motor, and cognitive tasks

Duration and frequency of substance use values were extracted from the modified Lifetime Drug Usage Questionnaire^30–33^ (see Table 3). Duration of use was measured in years as the sum of all phases during which the participant was actively using a given substance. Frequency of use was measured on a five-point scale, wherein during an active phase: 1 = used at least once annually, 2 = used at least once monthly, 3 = used at least once weekly, 4 = used daily or near-daily, and 5 = used multiple times daily. We computed a weighted frequency of use by drug as the product of frequency of use (5-point scale) x phase duration (years), divided by the aggregate of all phases associated with that drug (years).

**Table 3:** Stimulant and opioid variables across groups.

	HIV−/POLY− (*n* = 34)	HIV−/POLY + (*n* = 27)	HIV + /POLY− (*n* = 17)	HIV + /POLY + (*n* = 15)	*P*-value
Methadone dose (mg) (SD)	–-	85.15 (32.3)* n* = 19	–-	87.50 (24.8) *n* = 2	0.917
Suboxone dose (mg) (SD)	–-	13.60 (6.7) *n* = 5	–-	12.00 (5.7) *n* = 2	0.776
DOA stimulants (y) (SD)	23.00 (12.7) *n* = 9	6.39 (8.3) *n* = 23	13.50 (17.7) *n* = 2	12.47 (7.4)	**0.004**^A-B^, 0.583^A-C^, **0.044**^A-D^, 0.671^B-C^, **0.025**^B-D^, 0.874^C-D^
DOU stimulants (y) (SD)	3.17 (7.7)	15.29 (12.7)	1.00 (3.6)	17.53 (9.5)	** < 0.001**^A-B^, 0.179^A-C^, < **0.001**^A-D^, < **0.001**^B-C^, 0.522^B-D^, < **0.001**^C-D^
FOU stimulants (y) (SD)	0.40 (0.8)	2.97 (1.3)	0.18 (0.5)	3.06 (1.3)	** < 0.001**^A-B^, 0.272^A-C^, < **0.001**^A-D^, < **0.001**^B-C^, 0.835^B-D^, < **0.001**^C-D^
DOA opioids (y) (SD)	–-	2.46 (2.6) *n* = 24	–-	10.09 (6.4) *n* = 11	** < 0.001**
DOU opioids (y) (SD)	–-	16.99 (9.9)	–-	11.80 (9.9)	0.193
FOU opioids (y) (SD)	–-	3.34 (1.2)	–-	2.64 (1.8)	0.134

Statistics are reported as in Table 1. Post-hoc comparisons included: A = HIV-/POLY-, B = HIV-/POLY + , C = HIV + /POLY-, D = HIV + /POLY + . DOA = duration of abstinence, DOU = duration of use, FOU = frequency of use, SD = standard deviation, mg = milligram, y = years. Significant values are in [bold], *p* < 0.05.

Independent samples *t* tests were used on normally distributed continuous variables. ANOVAs were used to compare three or more groups. Mauchly’s Test of Sphericity was applied to mixed-design ANOVAs and, when the assumption of sphericity was violated, a Greenhouse–Geisser correction was applied. Post-hoc Games-Howell tests were used following mixed-design ANOVAs that contained unequal sample sizes and variance. Pearson Chi-Square tests were used to compare categorical variables. Levene’s Test for Equality of Variances was used to identify unequal variances. Kruskal–Wallis H tests were used to compare ordinal data that did not follow a normal distribution or one-way ANOVAs with unequal variance, followed by post-hoc pairwise comparisons. Post-hoc Mann–Whitney tests were corrected for multiple comparisons (Bonferroni). Statistics were performed using IBM SPSS Statistics, Macintosh, version 27.0 (IBM Corp., Armonk, NY, USA).

## Results

### Cognitive and motor task results

#### Cognitive task

**Figure 1:**
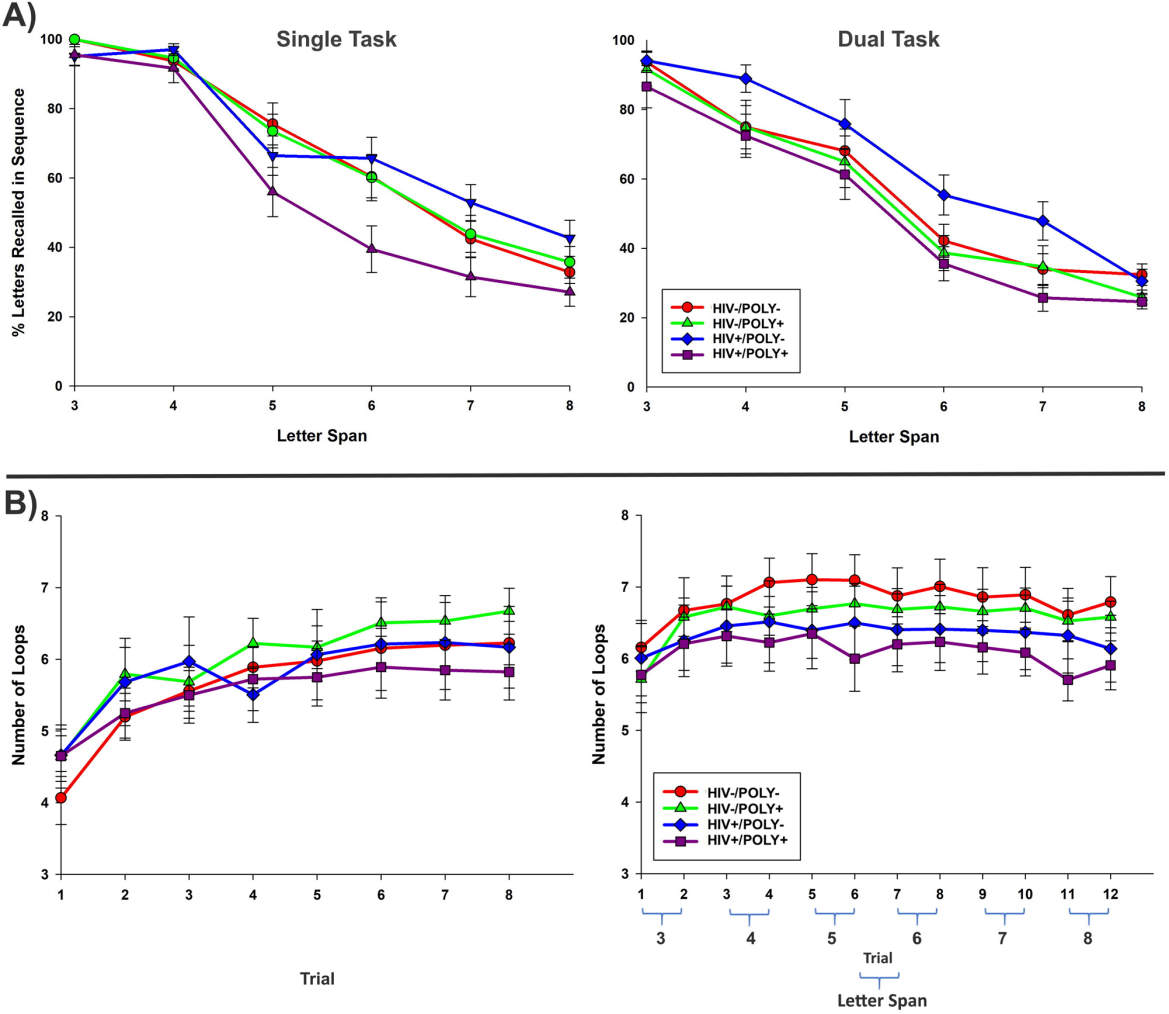
Working memory and loop drawing performance during single and dual task conditions**. **(**A**) The percent accuracy of letter spans recalled in sequence are reported across trials of increasing letter spans of 3–8 letters. Recall accuracy for letters decreased as the size of letter spans increased, especially in the dual task condition when working memory was performed in conjunction with a motor task. In the single task condition, the HIV + /POLY + group (purple) performed with less accuracy than did the HIV + /POLY- group (blue). (**B**) The number of loops is reported across each 5-s trial. Loop drawing frequency increased across trials, with most gains occurring in the single task condition. In the dual task condition, loop drawing was done in conjunction with a working memory task that increased cognitive load across trials. The HIV-/POLY- group (red) improved motor learning more than any other group did, suggesting that HIV and substance use history impacted performance. However, when HIV and POLY groups were examined collectively, the HIV + groups (blue plus purple) showed impaired motor learning, whereas the POLY + groups (green plus purple) did not. (Trials 9–12 of the dual task were not included in omnibus analysis). Bars denote one standard error.

A mixed-design ANOVA was conducted with 6 (span: 3–8 letters) × 2 (condition: single vs. dual task) as within-subjects factors and 4 (group: HIV-/POLY-, HIV-/POLY + , HIV + /POLY-, and HIV + /POLY +) as a between-subjects factor (Fig. 1A). Results revealed an interaction of condition x group, *F*(3, 58) = 3.13, *p* = 0.032. Post-hoc tests indicated that the HIV + /POLY + group performed less accurately than did the HIV + /POLY- group in the single task condition (See Supplement 1 for additional results).

#### Motor task

A mixed-design ANOVA was conducted with 8(trial: trials 1–8) × 2(condition: single vs. dual) as within-subjects factors and 4(group) as a between-subjects factor (Fig. 1B). Only the first eight trials of the dual task were included because we could not load an uneven number of trials into the ANOVA. Results revealed an interaction of trial x group, *F*(21, 392) = 2.02, *p* = 0.005 (See Supplement 1for additional results). Post-hoc tests indicated that the HIV-/POLY- showed a greater increase in loop drawing across trials (i.e., motor learning) than did all other groups. The remaining groups did not differ from one another.

To investigate how the HIV + groups performed regardless of substance dependence history, we conducted a mixed-design ANOVA with 8(trial) × 2(condition) as within-subjects factors and 2(group: HIV- (collapsed across POLY- and POLY +) and HIV + (collapsed across POLY- and POLY +)) as a between-subjects factor. There was an interaction of trial x group, *F*(7, 406) = 5.01, *p* < 0.001, indicating that the HIV + group showed a disproportionately slow rate of motor improvement across trials (See Supplement 1 for additional analyses). We also examined how POLY + groups performed regardless of HIV status. We conducted a mixed-design ANOVA with 8(trial) × 2(condition) as within-subjects factors and 2(group: POLY- (collapsed across HIV- and HIV +) and POLY + (collapsed across HIV- and HIV +) as a between-subjects factor. There was no main effect of group or additional 2-way or 3-way interactions, all *F* < 0.80, *p* > 0.38. Thus, groups did not differ in their motor performance as a function of substance dependence history. Taken together, these results indicated that HIV had a stronger impact on motor performance than did substance use.

### Voxel-based morphometry results

To investigate changes in whole-brain GM volume, we ran statistical analyses between clinical groups and controls. A Levene's test for equality of variance indicated unequal variances between the groups, *F*(3,89) = 4.48, *p* = 0.006. When the Kruskal–Wallis test revealed that whole-brain GM significantly differed among groups, *H*(3) = 11.5, *p* = 0.009, post-hoc tests compared all pairs of groups. The whole-brain GM volumes of the HIV + /POLY- (*Mdn* = 0.42) and HIV + /POLY + (*Mdn* = 0.44) groups were smaller than that of controls (*Mdn* = 0.46) [Controls vs. HIV + /POLY-: *U* = 21.2, Bonferroni correction *p* = 0.048; Controls vs. HIV + /POLY + : *U* = 22.6, Bonferroni correction *p* = 0.042]. None of the other comparisons were significant (all *p*-values > 0.31).

**Figure 2:**
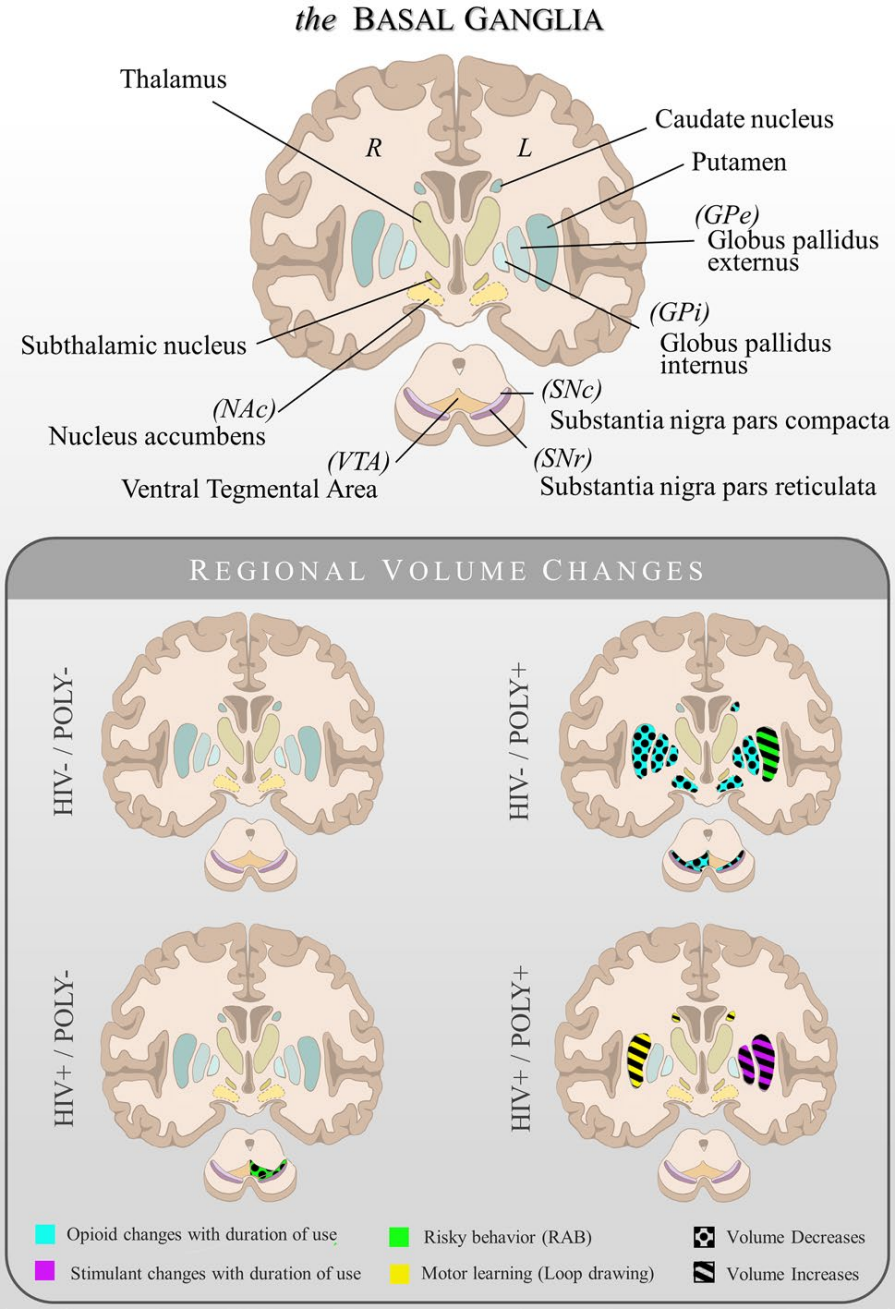
Summary of gray matter volume changes within the basal ganglia for each study group**.** Top: A coronal view schematic of the regions of interest examined within the basal ganglia and midbrain. L = left; R = right. Bottom: Colored regions indicate the locations within the basal ganglia that were associated with duration of substance use, motor learning, and risky behaviors. Patterns indicate the positive or negative direction of the association. Depictions may be compared with Fig. 3 and Table 4.

We then conducted regressions between BG ROIs GM volumes and performance on the cognitive and motor tasks (See Fig. 2). For the cognitive task, we selected each participant’s mean score on the 5-letter span trial during single and dual task conditions because this span contained the highest cognitive load before group differences emerged (confirmed by one-way ANOVAs for each span length within each condition). For single and dual task cognitive paradigms, there were no significant associations between BG ROI GM volumes and cognitive performance (all p-values > 0.05 FWE). For the motor task, we entered the Δ between single task trials 8 and 1 into a regression analysis with BG ROI GM volumes for each condition. The HIV + /POLY + group revealed a positive association in GM volume bilaterally in the caudate and right putamen (*p* < 0.05 FWE) with greater motor learning (higher Δ) (Table 4). There were no further significant volume associations between other BG regions regarding motor learning (all *p*-values > 0.05 FWE).

**Table 4:** Regressions between regional basal ganglia gray matter volume, motor learning, pattern of substance use, and risky behavior.

	Group	t-value	Cluster size (voxels)	MNI peak coordinates (x, y, z)	ROI mask location
**Motor learning**					
Loop drawing (Δ trials 1 -8)	HIV + /POLY +	7.15	15	− 12, 22, 6	Caudate (L)
		9.98	43	14, 21, 10	Caudate (R)
		6.19	23	30, 8, 2	Putamen (R)
**Substance use duration**					
Opioids (years)	HIV-/POLY +	− 4.52	119	22, − 2, − 2	GPe (R)
		− 4.40	80	2, − 16, − 4	SNc (R)
		− 4.78	68	15, − 2, 3	GPi (R)
		− 3.86	67	15, 6, − 15	NAc (R)
		− 3.89	59	− 15, 2, − 10	GPe (L)
		− 4.60	59	− 6, 6, − 12	NAc (L)
		− 4.43	17	− 12, 4, 6	Caudate (L)
		− 4.06	14	21, 2, − 6	Putamen (R)
		− 3.53	11	− 21, − 3, − 2	GPi (L)
		− 3.72	10	2, − 15, − 15	VTA (R)
Stimulants (years)	HIV + /POLY +	6.34	17	− 27, − 4, 6	GPe (L)
		7.12	5	− 28, − 3, 8	Putamen (L)
**Substance use frequency**					
Opioid weighted frequency (scaled frequency)	HIV-/POLY +	− 4.15	89	− 24, − 4, 0	GPe (L)
		− 3.98	41	− 21, − 6, 2	GPi (L)
		− 4.96	40	− 3, 6, − 8	NAc (L)
		− 2.99	17	15, 10, − 15	NAc (R)
**Risky behavior**					
RAB* (total score)	HIV-/POLY +	7.18	6	− 18, 10, − 3	Putamen (L)
	HIV + /POLY-	− 5.64	53	− 3, − 20, − 9	SNc (L)
		− 3.30	8	2, − 18, − 16	VTA (L)

Results met criteria for *p* < 0.05 FWE with a cut-off threshold of ≥ 5 voxels. Negative t-values represent negative associations. L = left; R = right.

Groups were compared on total BG GM differences, controlling for demographic variables, total brain GM, and TCV. Results indicated that total BG GM was reduced in all three clinical groups, relative to that of controls, *p* < 0.005 (vs. HIV + /POLY-, *p* < 0.005; vs. HIV-/POLY + and HIV + /POLY + , *p* < 0.001). However, none of these differences met criteria for significance at *p* < 0.05 FWE. Examination of BG ROIs revealed a left-lateralized reduction of GM volume in the clinical groups (Table 5). Compared to controls, the HIV-/POLY + group showed GM reductions in the left GPe, left GPi, and left caudate. In the HIV + /POLY + and HIV + /POLY- groups, GM reduction was observed in the left GPe (See Supplement 1 for additional group differences with clusters < 5 voxels and *p* < 0.05 FWE).

**Table 5:** Gray matter ROI volume differences between controls versus clinical groups within the basal ganglia.

Controls (HIV-/POLY−) vs. clinical groups	t-value	Cluster size (voxels)	MNI peak coordinates (x, y, z)	Location
**HIV-/POLY + **				
	− 3.69	96	− 18, 2, 2	GPe (L)
	− 3.87	92	− 18, − 2, 3	GPi (L)
	− 3.52	10	− 6, 8, 4	Caudate (L)
**HIV + /POLY + **				
	− 3.34	13	− 21, 4, 4	GPe (L)
**HIV + /POLY−**				
	− 3.28	10	− 20, 3, 3	GPe (L)

Data represent clusters with significant GM reduction in the basal ganglia in each clinical group relative to that of controls (*p* < 0.05 FWE with a cut-off threshold of ≥ 5 voxels) in each ROI within the basal ganglia. L = left; R = right.

**Figure 3:**
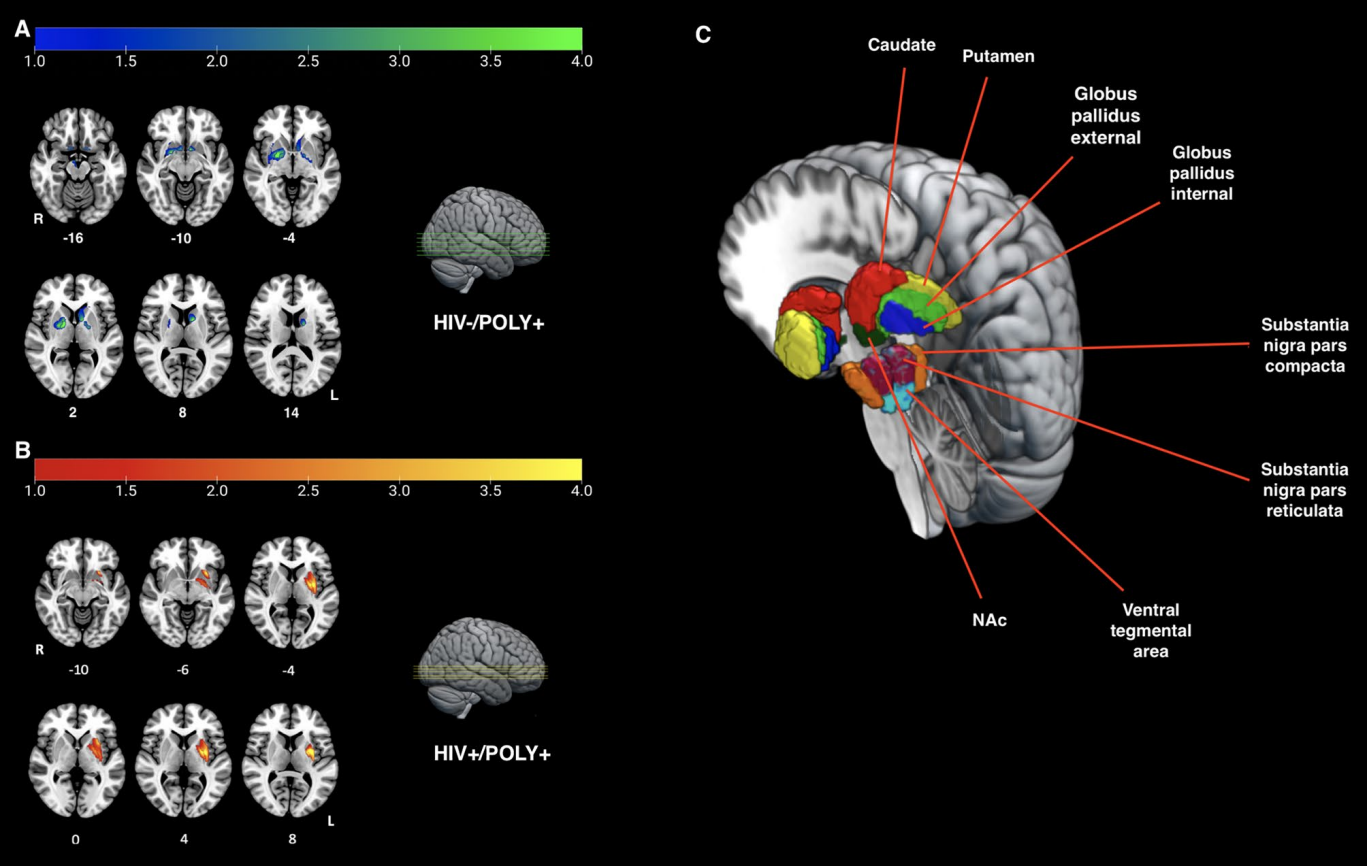
POLY + group regressions of basal ganglia gray matter volume and substance use duration. (**A**) Statistical map of z-scores indicating reduced gray matter (GM) as a function of opioid use duration in the HIV-/POLY + group; z-planes = -16, -10, -4, 2, 8, 14 in MNI space*; p* < 0.05 FWE. R = right hemisphere, L = left hemisphere; (**B**) Statistical map of z-scores indicating increased GM as a function of stimulant use duration in the HIV + /POLY + group; z-planes = -10, -6, -4, 0, 4, 8 in MNI space*; p* < 0.05 FWE. R = right hemisphere, L = left hemisphere. (**C**) Depiction of basal ganglia ROI masks that were used for regression analyses with substance use duration, shown here overlaid on the SPM152 template for visualization.

Regression analysis of within-group differences examined changes in BG GM ROI volumes as a function of duration and frequency of use for opioids and stimulants in the HIV-/POLY + and HIV + /POLY + groups (see Table 4 and Fig. 3). For the HIV-/POLY + group, GM volume reduction in the caudate, putamen, GP, SNc, NAc, and VTA was found in relation to prolonged opioid use after controlling for duration of stimulant use (Fig. 3A). Similarly, a reduction of GM volume was found in relation to opioid-weighted frequency of use, controlling for stimulant weighted frequency of use, in the left GPe, left GPi, and bilateral NAc (all results at *p* < 0.05 FWE) (Table 4). For the HIV + /POLY + group, an increase in GM volume was found in relation to prolonged stimulant use, controlling for duration of opioid use, in the left GPe and putamen (Fig. 3B). No further associations between other BG regions as a result of opioid or stimulant use and stimulant-weighted frequency of use were observed.

Total RAB and total BIS scores were entered into regression analyses within each group to examine associations between the BG GM volume and risk-associated behaviors (Table 4). The HIV-/POLY + group showed a positive association between RAB scores and GM in the left putamen (*p* < 0.05 FWE). The HIV + /POLY- group showed a negative association between RAB scores and GM in the left SNc and left VTA (all results *p* < 0.05 FWE). There were no further associations between RAB scores and BG ROIs within the groups (all *p* > 0.05 FWE). In addition, there were no associations between BIS scores and BG ROIs within the groups (all *p* > 0.05 FWE).

Additional ROI regression analyses within each group explored the following clinical variables to determine if GM changes were present: estimated duration of HIV infection (years), current CD4, nadir, and ART adherence (lifetime). Binary data were excluded from VBM analysis, such as AIDS diagnosis (CD4 < 200 cells/mm^3^), HIV viral load < 20 copies/mL, and EFV exposure. There were no associations between these variables and ROI GM volumes observed at *p* < 0.05 FWE with a cluster size of 5 or more voxels. We then lowered the statistical threshold to *p* = 0.001 uncorrected for further exploration. At this threshold, we observed a positive association between current CD4 levels and GM volume in the left caudate for the HIV + /POLY + group, *p* = 0.001. There were no other associations found at *p* = 0.001 or less.

## Discussion

Consistent with prior research, our observations indicated that HIV and polysubstance use impacted BG GM volumes^47–49^ We observed differential effects of polysubstance use on BG volume as a function of HIV infection. In HIV-/POLY + participants, duration and frequency of opioid use was associated with BG GM atrophy, but no effect was seen with the corresponding variables for stimulants (Fig. 2). By contrast, in HIV + /POLY + participants, duration of stimulant use was associated with BG GM enlargement, while no such effect was observed with opioid use. Taken together, these results suggest that the substance an individual preferentially uses on a regular basis may differentially impact BG GM volume contingent upon serostatus.

It is curious that the direction of volumetric BG change was predicated upon the duration of substance use and substance type (opioid versus stimulants) and that this effect was mediated by HIV. Although effects of opioids on the BG have not been reported previously, past research suggests an antagonistic effect between volume decreases and increases related to other substance use in the setting of HIV infection^22,50^. One explanation for this volumetric increase is neuroinflammation^28^, which can be beneficial (e.g., tissue regeneration, protection against infection) or detrimental (e.g., cell death, chronic neurodegeneration). Sil et al*.*^51^ hypothesized that HIV-1 proteins and drugs of abuse cooperatively activate inflammasomes, leading to neuroinflammation and contributing to premature aging. Glial cell activation and subsequent cerebral scarring have been noted as a hallmark of neuroinflammation in the context of HIV^52^. Stimulants have been associated with microglial activation and reactive astrogliosis within the dopaminergic system^51,53^, compounding the neurotoxic effects of HIV on dopamine-related functions^54,55^ and increasing pro-inflammatory cytokines^56–58^. We speculate that the observed BG volume increases in PLWH resulted from a stimulant-induced inflammatory response that was disproportionate to the smoldering, degenerative milieu typical of HIV infection^59,60^.

We also observed whole-brain GM volume decrements in the HIV + groups relative to controls, mirroring previous research^61–63^ and suggesting that there were GM changes in regions outside of the BG. Therefore, we included whole-brain GM as a covariate in our between-group comparisons of the ROIs. Relative to controls, each clinical group revealed regional BG GM volume decreases, although these findings did not meet the stringent threshold of *p* < 0.05 FWE. This may have been a statistical power issue. Tentatively, we interpreted these findings indicative of BG GM volume decreases that surpassed GM volume changes at the whole-brain level, speaking to the particular vulnerability of the basal ganglia in these populations.

The associations we observed between the BG GM and risky behaviors reinforced the expected outcomes of BG insult. The HIV-/POLY + group showed an increased BG GM volume (putamen) in association with increased risk-taking behavior. This finding aligns with past observations linking increased striatal response on fMRI to reward cues and anticipation of potential gain^64,65^. In contrast, the HIV + /POLY- group showed the opposite effect, with volume reductions in the SNc and VTA associated with more risk-taking behavior (See Fig. 3). HIV seropositivity has been linked with higher risk-taking scores on the RAB^66^, and we speculate that neuronal atrophy in these dopaminergic regions^67,68^ may be implicated in this association. We also observed motor deficits in the clinical groups, with the HIV + /POLY + group showing an association between decreased BG GM (caudate and putamen) and slower motor learning. This finding is unsurprising given the BG’s prominent role in motor learning and control^69,70^.

Analysis of clinical variables indicated that an increase of GM volume was present in the left caudate in association with increased current CD4 count in the HIV + /POLY + group. There were no other associations found within groups or between groups after exploring other clinical variables. Our findings are similar to those of Castelo et al.^71^, who demonstrated higher CD4 counts were associated with larger left putamen volume and Fennema-Notestine et al.^72^, who observed higher CD4 counts were associated with an increase in subcortical GM. Although the relationship is complex, Fennema-Notestine et al. suggested that CD4 counts reflect immune recovery processes following GM changes.

Despite controlling for substance use in our regression analyses, we cannot fully dissociate the independent contributions of stimulants and opioids in our samples or determine the influence, if any, of opioid maintenance therapies on BG GM volume. In this study, all participants in the HIV-/POLY + group and four participants in the HIV + /POLY + group were receiving opioid maintenance therapy at the time of test. While methadone has been shown to increase viral load^73^ and contribute to neuroinflammation and viral neuropathogenesis^60^, buprenorphine increases the likelihood of viral suppression in PLWH^74^. Increased dopamine concentrations related to opioid use, including methadone, can further increase monocyte influx and viral replication^75,76^. It is possible that opioid therapy influenced the volumetric changes we observed in both POLY + groups; however, differences were found in opposite directions between them. We also cannot exclude the influence of ART on GM volume, as it may interact with BG immune responses^77–79^. However, HIV + participants in both groups were adherent to an ART regimen at the time of testing, and we observed a GP volume increase in the HIV + /POLY + group only. Finally, the cross-sectional design of this study presents a limitation to our understanding of the evolving impact of substance use and HIV on the brain.

We observed that the integrity of the basal ganglia was affected by the interaction between HIV and polysubstance use. Combined HIV infection and stimulant use may lead to sustained neuroinflammation and damage to the BG, as evidenced by pathological GM volume increases. These results shed light on structural brain changes associated with HIV infection and polysubstance exposure, which play a critical role in understanding clinical implications for PLWH. Further research is warranted to determine the precise underlying neurological mechanisms of BG changes and why it is particularly vulnerable to these effects.

## Supplementary Information

Supplementary Information.

## Data Availability

The datasets generated during and/or analyzed during the current study are available from the corresponding author on reasonable request.
