# Supplementary material for: Characterization of basal ganglia volume changes in the context of HIV and polysubstance use
Source: Sci Rep. 2022 Mar 14;12:4357. doi: 10.1038/s41598-022-08364-0 (PMC8921181; doi:10.1038/s41598-022-08364-0)
Supplement: Supplementary file 1 — Supplementary Information. [file 41598_2022_8364_MOESM1_ESM.docx]

**Supplement 1: Motor and Cognitive Tasks**

**METHODS**

Participants performed a motor-only task [HIV-/POLY- (*n* = 17), HIV-/POLY+ (*n* = 14), HIV+/POLY- (*n* = 17), HIV+/POLY+ (*n* = 15)], a cognitive-only task [HIV-/POLY- (*n* = 16), HIV-/POLY+ (*n* = 14) , HIV+/POLY- (*n* = 17), HIV+/POLY+ (*n* = 15)], and a cognitive-motor combined dual task [HIV-/POLY- (*n* = 16), HIV-/POLY+ (*n* = 13), HIV+/POLY- (*n* = 17), and HIV+/POLY+ (*n* = 15)].

In the motor-only task, participants were asked to draw as many complete figure-8 loops as possible over a 5-second duration over eight repeated trials, with 3-second breaks between trials. In the cognitive-only task, participants were asked to rehearse a sequence of three to eight consonant letters read aloud by the experimenter for 12 trials. After every two trials, the letter span increased by one additional consonant letter. Participants were then asked to recall the letters following a 5-second period of silent rehearsal. The cognitive-motor dual task began with the participant hearing a sequence of three to eight consonant letters. As soon as the letters were heard, participants drew figure-8s while mentally rehearsing the letter sequence for five seconds. The participant was then asked to recall the letters in sequence (total of 12 trials).

Motor data were recorded using a Wacom Intuos 13” tablet (48.7 x 31.8 x 1.2 cm), non-marking stylus, and MovAlyzeR software (Neuroscript LLC, Tempe, AZ, USA). One HIV+/POLY- participant was excluded from the motor-only task analysis due to a software error during recording. Figure-8 loop counts were scored by two independent raters for consensus. Letter sequence accuracy was scored by counting the total number of letters recalled in sequence, divided by the total number of letters in the sequence (reported as a percentage).

**RESULTS**

Cognitive Task

Results revealed main effects of span, *F*(4.15, 241) = 237, *p* < .001, condition, *F*(1, 58) = 59.8, *p* < .001, and group, *F*(3, 58) = 3.55, *p* = .02. There were also interactions of span x condition *F*(5, 290) = 4.92, *p* < .001 and condition x group, *F*(3, 58) = 3.13, *p* = .032. (see Figure 1A) These results indicated that recall accuracy for letter sequences declined with increasing letter spans, especially in the dual task condition. To determine the source of the condition x group interaction, 6(span) x 2(group) mixed-design ANOVAs were conducted within each condition. This yielded a marginally significant span x group interaction in the single condition, *F*(11.8, 228) =1.78, *p* = .055, with no significant interaction in the dual condition, *F*(12.6, 243) = .634, *p* = .819. A post-hoc Games-Howell test was used to determine group differences within the single task condition, yielding a marginal group difference between the HIV+/POLY- and HIV+/POLY+ groups (mean difference = 13.12), *p* = .056 and no other significant group differences, all *p* > .112. Thus, the HIV+/POLY+ group performed less accurately than did the HIV+/POLY- group in the single task condition. There was no 3-way interaction of span x condition x group, *F*(15, 290) = 1.05, *p* = .41.

Motor Task

To examine motor performance, a mixed-design ANOVA was conducted with 8(trial: trials 1-8) x 2(condition: single vs. dual) as within-subjects factors and 4(group: HIV-/POLY-, HIV-/POLY+, HIV+/POLY-, and HIV+/POLY+) as a between-subjects factor. Only the first eight trials of the dual task were included because we could not load an uneven number of trials into the ANOVA. Results revealed an interaction of trial x group, *F*(21, 392) = 2.02, *p* = .005. Results revealed main effects of trial, *F*(3.09, 173) = 62.9, *p* < .001, and condition, *F*(1, 56) = 44.5, *p* < .001. There were also interactions of trial x condition, *F*(3.90, 218) = 13.8, *p* < .001, and trial x group, *F*(21, 392) = 2.02, *p* = .005. (see Figure 1B) These results indicated that participants increased their loop drawing across trials, with a steep increase during the single task, and a higher number of loops drawn in the dual task overall. To determine the source of the trial x group interaction, 16(trials 1-8 of both conditions) x 2(group) mixed-design ANOVAs were conducted on every possible group pairing. This yielded significant trial x group interactions between the HIV-/POLY- group (controls) versus all other groups [vs. HIV-/POLY+, *F*(15, 450) = 2.24, *p* = .005; HIV+/POLY-, *F*(15, 450) = 2.43, *p* = .002; HIV+/POLY+, *F*(15, 435) = 2.60, *p* = .001]. All other trial x group pairings were non-significant, all *F* < 1.06, *p* > .39. Thus, the HIV-/POLY- showed a greater increase in loop drawing across trials than did all other groups, while the other groups did not differ from each other in this regard. There was no main effect of group, *F*(3, 56) = .85, *p* = .47, or 3-way interaction of trial x condition x group, *F*(21, 392) = .55, *p* = .95.

To investigate how groups performed with respect to HIV status (regardless of substance use history), we conducted a mixed-design ANOVA with 8(trial) x 2(condition) as within-subjects factors and 2(group: HIV- (collapsed across POLY- and POLY+) and HIV+ (collapsed across POLY- and POLY+)) as a between-subjects factor. The original main effects of trial and condition and the interaction of trial x condition were replicated. There was no main effect of group, *F*(1, 58) = 2.52, *p* = .12, or 3-way interaction of trial x condition x group, *F*(7, 406) = .69, *p* = .68. There was an interaction of trial x group, *F*(7, 406) = 5.01, *p* < .001. Post-hoc trial x condition comparisons within each group yielded main effects of trial [HIV-, *F*(2.62, 73.4) = 45.3, *p* < .001; HIV+, *F*(3.37, 101) = 20.2, *p* < .001], which made it difficult to determine the source of the interaction. Therefore, we compared groups on the difference (delta) in number of loops drawn between trial 1 of the single task and trial 8 of the dual task. An independent samples t-test indicated that the delta was greater for HIV- (*M* = 2.55, *SD* = 1.61) than for HIV+ (*M* = 1.67, *SD* = 1.19), *t*(60) = 2.43, *p* =.018. Thus, the HIV+ group showed a slowed rate of motor improvement across trials.

**Supplementary Table S1.** Between groups and regression analysis on basal ganglia gray matter ROIs and pattern of substance use.

|  | | | **Group** | **t-value** | **Cluster Size  (voxels)** | **MNI Coordinates (x, y, z)** | **Location** |
| --- | --- | --- | --- | --- | --- | --- | --- |
| **Controls (HIV-/POLY-)**  **vs. Clinical Group**^A^ | | | HIV-/POLY+ | 3.91 | 3 | -16, 2, 2 | GP (L) |
| **Opioids** (years) | | | HIV-/POLY+ | -3.86 | 2 | -20, 2, -10 | Putamen (L) |
| **Opioid weighted frequency** (scaled frequency) |  |  | HIV-/POLY+ | -4.06 | 2 | -6, 6, -8 | Caudate (L) |
| **Stimulant weighted frequency** (scaled frequency) | | | HIV-/POLY+ | 3.74 | 2 | 16, 15, 6 | Caudate (R) |

A = Between group analysis of greater gray matter regions in controls versus clinical groups.
Covariates include age, sex, education, handedness for between group and regression analysis. Negative t-values represent negative correlations. Significant values reported at p < 0.05 FWE; L = left; R = right.
